# Supplementary figures and images for: Genome-wide characterization and expression analysis of the CONSTANS-like gene family of Juglans mandshurica Maxim
Source: PeerJ. 2025 Apr 18;13:e19169. doi: 10.7717/peerj.19169 (PMC12011014; doi:10.7717/peerj.19169)

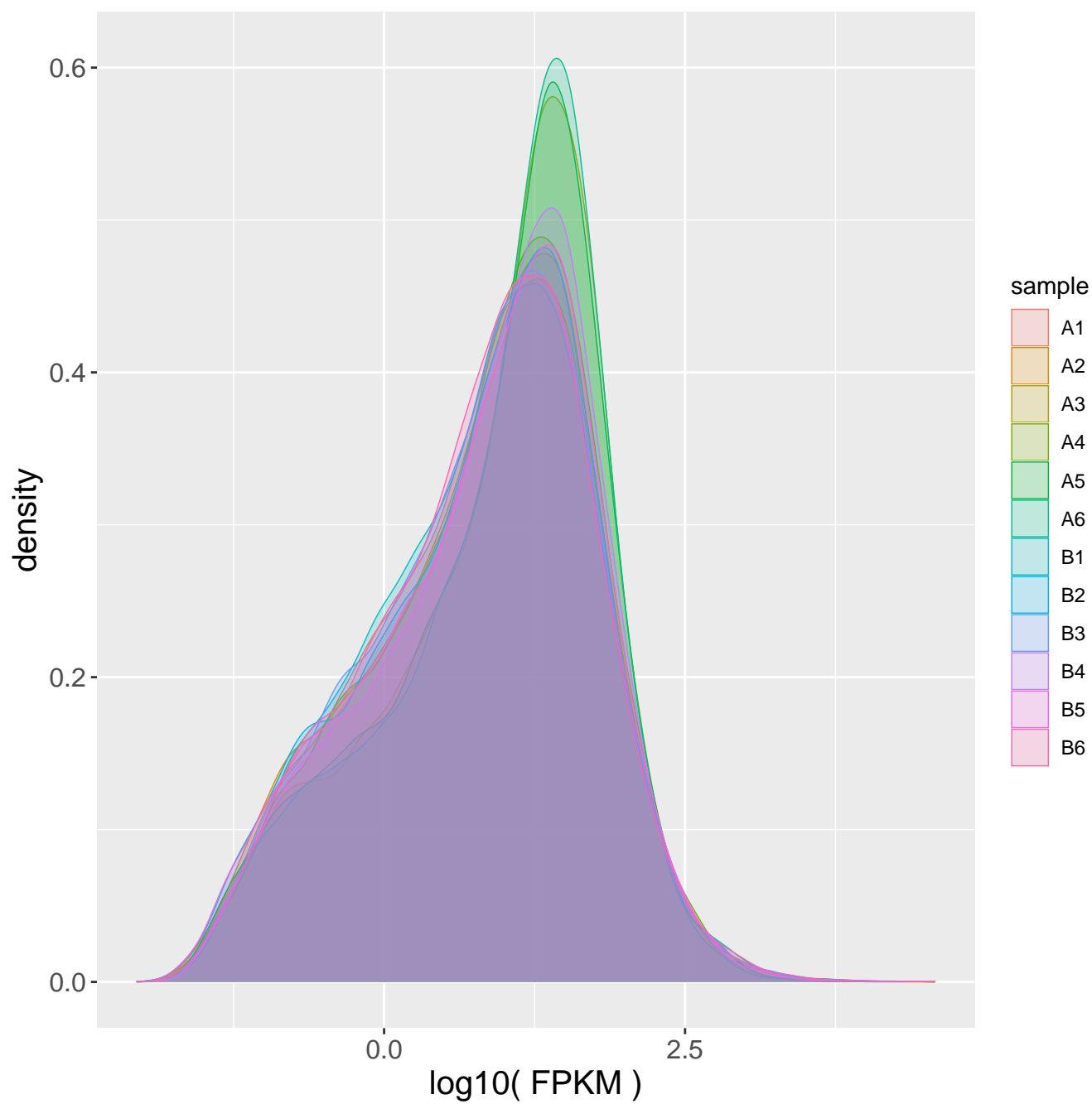

Supplement: Supplemental Information 3 [file peerj-13-19169-s003.pdf]
